# Detection of brain network abnormalities by graph invariants in Alzheimer’s disease using MRI images

**DOI:** 10.1038/s41598-025-26259-8

**Published:** 2025-11-26

**Authors:** G. NallappaBhavithran, R. Selvakumar

**Affiliations:** Department of Mathematics, School of Advanced Sciences, Vellore Institute of Technology, Vellore, 632014 Tamil Nadu India

**Keywords:** Topological indices, Neural network, WS model, Brain graph, Small-world networks., Computational neuroscience, Machine learning, Network topology

## Abstract

Alzheimer’s disease is a major cause of dementia in older adults. It involves gradual changes in brain function that result in cognitive decline, affecting memory, reasoning, and executive skills. The accurate detection of structural abnormalities in brain networks is crucial for early diagnosis and disease staging. This study presents a graph-based framework that analyzes abnormalities in brain networks of Alzheimer’s patients using six distance-based topological indices: Szeged index, Graovac-Ghorbani index, Padmakar–Ivan index, Mostar index, Wiener index, and Normalized Graovac-Ghorbani index. These indices effectively characterize the structural properties of brain networks and identify disruptions linked to disease progression. The proposed framework first constructs brain graphs from MRI images using the Brightness Distance Matrix method, which captures the spatial relationships between pixels. Then, the constructed brain graphs are modeled using the Watts and Strogatz small-world model to normalize the topological indices. The normalized indices serve as input features for various machine learning models, including decision trees, logistic regression, support vector machines, and a multi-layer neural network. Among these models, a refined neural network model achieves the highest classification accuracy of 89.45%, confirming the value of topological indices as interpretable biomarkers for disease staging. This framework demonstrates the potential of graph-theoretic approaches for detecting Alzheimer’s-related brain network alterations and offers a scalable, interpretable, and privacy-friendly solution.

## Introduction

Dementia is a progressive brain disorder marked by memory loss, cognitive decline, and difficulties in daily activities. It is influenced by several factors, including changes in brain structure and function. Although its causes and risk factors have been widely studied, how different brain regions and their connections affect dementia progression remains an active area of research. Since different parts of the brain support various cognitive functions, the connections between them are essential for maintaining overall brain health.

The key brain regions that are affected by dementia include the hippocampus, Medial Temporal Lobe (MTL), white matter, and Default Mode Network (DMN). The MTL, which encompasses the hippocampus, entorhinal cortex, and parahippocampal regions, plays a central role in memory encoding, storage, and retrieval. Among these, the hippocampus is crucial for memory formation and retrieval, and it is often one of the first areas affected by Alzheimer’s Disease (AD), a common cause of dementia^1^. White matter which is composed of myelinated nerve fibres, enables communication between different brain areas. Damage to these fibres, such as demyelination or axonal injury, can disrupt neural connections and increase the risk of dementia^2^. The DMN is a network of brain regions, active during rest and involved in self-reflection and internal thought processes. Disruptions in this network have been associated with AD and are thought to contribute to cognitive decline. The impact of AD has been investigated across different populations using various diagnostic methods and imaging techniques^3–5^, with advancements in machine learning (ML) approaches significantly improving early detection. Traditional ML algorithms, such as Support Vector Machines (SVM), Decision Trees, and Random Forests, have shown good performance in identifying potential AD cases^6–9^.

Early approaches in AD classification relied heavily on manual feature extraction from imaging data. Commonly used techniques includes histogram-based feature extraction and the calculation of image-derived statistical features. For instance, one study extracted histogram-based features from MRI images and utilized Neighborhood Component Analysis (NCA) for feature selection, successfully classifying the four stages of AD with high accuracy^10^. Another method analyzed the eigenvalues derived from the inertia tensor matrix of MRI images, reaching up to 90% accuracy in staging AD progression^11^. While these technique are effective, they often depend on carefully designed features and may struggle with high-dimensional data and subtle image variations.

To address these challenges, a deep triplet network was proposed to improve robustness and generalizability in AD detection^12^. In addition to that, a study successfully analyzed 3D MRI images using Convolutional Neural Networks (CNNs), achieving impressive accuracy rates by capturing spatial and temporal dependencies within the data^13^. For more complex representations, a deep Convolutional Autoencoder (CAE) architecture was introduced to perform non-linear decomposition of large-scale datasets^14^. This model enabled the visualization of influential brain regions and correlated structural features with factors such as age and protein deposits, offering a deeper understanding of cognitive decline. Another study employed a vision transformer-based architecture to analyze MRI images, achieving high accuracy in classifying AD stages^15^. Despite their strengths, deep learning models need a lot of annotated data and can overfit when only limited data is available. They also require significant computing power.

To overcome these limitations, recent studies have explored graph-based approaches for analyzing brain connectivity patterns. Emerging biomarkers from imaging data have provided valuable insights into the relationship between structural damage and cognitive function^16^. Functional Magnetic Resonance Imaging (fMRI), in particular, has been instrumental in evaluating the brain’s functional organization. The fMRI-based ML models have successfully distinguished patients with anxiety disorders from healthy individuals by analyzing graph-derived features and connectivity patterns^17,18^. Similarly, graph-based analyses of fMRI data have revealed key dynamic parameters, such as mean, variance, and kurtosis, which aid in identifying epileptogenic regions in temporal lobe epilepsy^19^. These findings suggest that network-based features can effectively capture disease-related changes in brain structure and function, particularly in conditions where annotated datasets are limited.

This paved the way for the application of graph-based techniques in addition to the Image based approaches. For instance, a study by Dogan et al. employed graph-theoretic approaches to map brain connectivity patterns using EEG signal data^20^. This work well demonstrated the potential of network analysis in capturing functional brain organization. However, EEG signals offer high temporal but limited spatial resolution, restricting the ability to detect localized structural changes within brain networks. Moreover, the study primarily focused on functional connectivity patterns, without addressing structural alterations, which are critically associated with the progression of neurodegenerative diseases such as AD. Nevertheless, this research highlighted the growing potential of combining network analysis with non-invasive, real-world digital biomarkers to improve early detection and monitoring of cognitive decline. A similar work on multi-modal sensors combined with federated learning algorithms have been implemented to monitor individuals in their natural environments for early signs of cognitive impairment^21^. These developments highlight the growing potential of combining network analysis with non-invasive, real-world digital biomarkers to improve early detection and continuous monitoring of cognitive decline. Similar graph-based deep learning frameworks have also been explored for other neurodegenerative diseases such as Parkinson’s Disease (PD)^22^. As a result, this study aims to improve the early detection of AD by analyzing alterations in brain network connectivity.

Early detection of AD remains crucial, particularly during its mild and moderate stages when diagnosis is often difficult. As dementia progresses, memory loss and cognitive decline are closely associated with weakened connectivity between different brain regions. This highlights the need for automated systems capable of analyzing topological changes in brain networks and identifying disrupted neural connections, which could support early-stage diagnosis and intervention. Although significant progress has been made in applying machine learning and deep learning techniques to AD detection, most existing models primarily rely on image intensity features, voxel-based analysis, or high-dimensional imaging data. While effective in many cases, these approaches often overlook the brain’s underlying structural organization and network-level interactions. However, that neurodegenerative diseases, including AD, are closely associated with changes in the brain’s topological structure and network dynamics. This highlights the need for network-based analysis in dementia research, as understanding these interactions could lead to more accurate detection and better insights into the disease’s progression.

Olaf Sporns is one of the pioneer to introduce graph theory to brain networks. In his work^23,24^, he introduced the term ‘connectome’ to describe the adjacency matrix representing brain connectivity. To study the connectome, it is essential to construct a reliable representation of the brain’s connectivity. Various methods, such as fMRI, parcellation, and the Brightness Distance Matrix (BDM), are commonly used to achieve this. Among these methods, BDM has emerged as a practical approach for constructing brain connectivity graphs from structural MRI data. Its ability to capture spatial relationships through pixel intensity distances makes it a useful tool for various network-based analyses. Bagheri et. al. have applied the BDM approach to brain MRI scans to construct connectomes and segment tumor regions using graph coloring techniques^25^. The main advantage of BDM lies in its ability to use the distances between pixel intensities to compute various statistical measures and image features, which are valuable for pattern recognition and classification tasks. Additionally, this method facilitates the identification of regions of interest (ROIs) and enables image segmentation, making it particularly effective for isolating specific anatomical structures or pathological areas within medical images. Another study by van Wijk et al.^26^ compared brain graphs of varying sizes and connection densities using the Watts-Strogatz (WS) small-world model. To reduce bias from empirical network structures, they first estimated empirical dependencies and then applied multiple thresholding techniques to construct and normalize brain graphs. Their work emphasized the importance of consistent normalization and parameter selection when comparing brain networks of different sizes and configurations.

In addition to constructing brain networks, examining their structural and topological properties is equally important. Brain graphs, like other real-world networks, exhibit a property called small-worldness. These small-world networks have high clustering coefficients like regular networks and low average path lengths like random networks, and they display unique dynamics not found in either regular or random networks. These networks with small-world properties can be studied by using a mathematical framework known as the WS model developed in 1998^27^. It has been used as a foundation for research on dynamics in various domains, including neuroscience, ecology, economics, and epidemiology. This model provides a way to generate random graphs that exhibit small-world characteristics, allowing researchers to analyze the structural and functional properties of real-world networks in a controlled manner.

The structural properties of a network can be characterized using various topological indices, which are numerical values that capture different aspects of the network’s organization. These indices can provide insights into the connectivity patterns, centrality, and overall structure of the network. In the context of brain networks, distance-based topological indices have been particularly useful in analyzing the connectivity and efficiency of brain graphs. These indices are derived from the shortest path distances between nodes in a graph and can reveal important information about the organization and function of neural networks. Gurunathan et. al used some of the distance based indices including Szeged, Padmakar–Ivan, and Mostar index to analyze centrality, peripherality, and other network characteristics in statistical cluster networks^28^. In another study, Ma et al.^29^ evaluated the effectiveness of various topological indices in measuring distances and supporting clustering tasks. To perform this study, they assigned a feature vector to each index and the utility of the index within specific classes of graphs. By comparing these indices against one another, the researchers determined the relative importance and discriminative power of each index within the corresponding graph family.

Despite the growing interest in using topological indices for graph analysis, neuroimaging classification methods still primarily rely on image intensity and voxel-level data. So, the examination of the brain’s connectivity patterns has not been thoroughly investigated. This study, therefore, aims to investigate whether distance-based topological indices, extracted from MRI-derived brain graphs, can effectively characterize alterations in brain connectivity. Furthermore, it examines the usage of the WS model in normalizing and interpreting these indices in the context of neurodegenerative network disruptions, and also evaluates whether machine learning classifiers trained on these graph-based topological indices can accurately distinguish between different stages of Alzheimer’s disease.

We also hypothesize that distance-based topological indices capture meaningful structural disruptions in brain connectivity patterns associated with AD, and that embedding these indices within a small-world network framework will enhance both the interpretability and classification performance of machine learning models applied for disease staging.

To provide a clear overview of the study’s structure, this paper is organized into four main sections. The first section presents a detailed literature review and highlights the novelty of the proposed model. The second section describes the construction of the WS model, introduces the path-based indices used in the study, and explains the conversion of MRI brain images into graphs using the BDM method. The third section evaluates the ability of six path-based indices to capture small-world properties, and reports the results of classifying different stages of Alzheimer’s disease using the proposed model. Finally, the fourth section summarizes the key findings, discusses limitations, and suggests directions for future work.

**Fig. 1:**
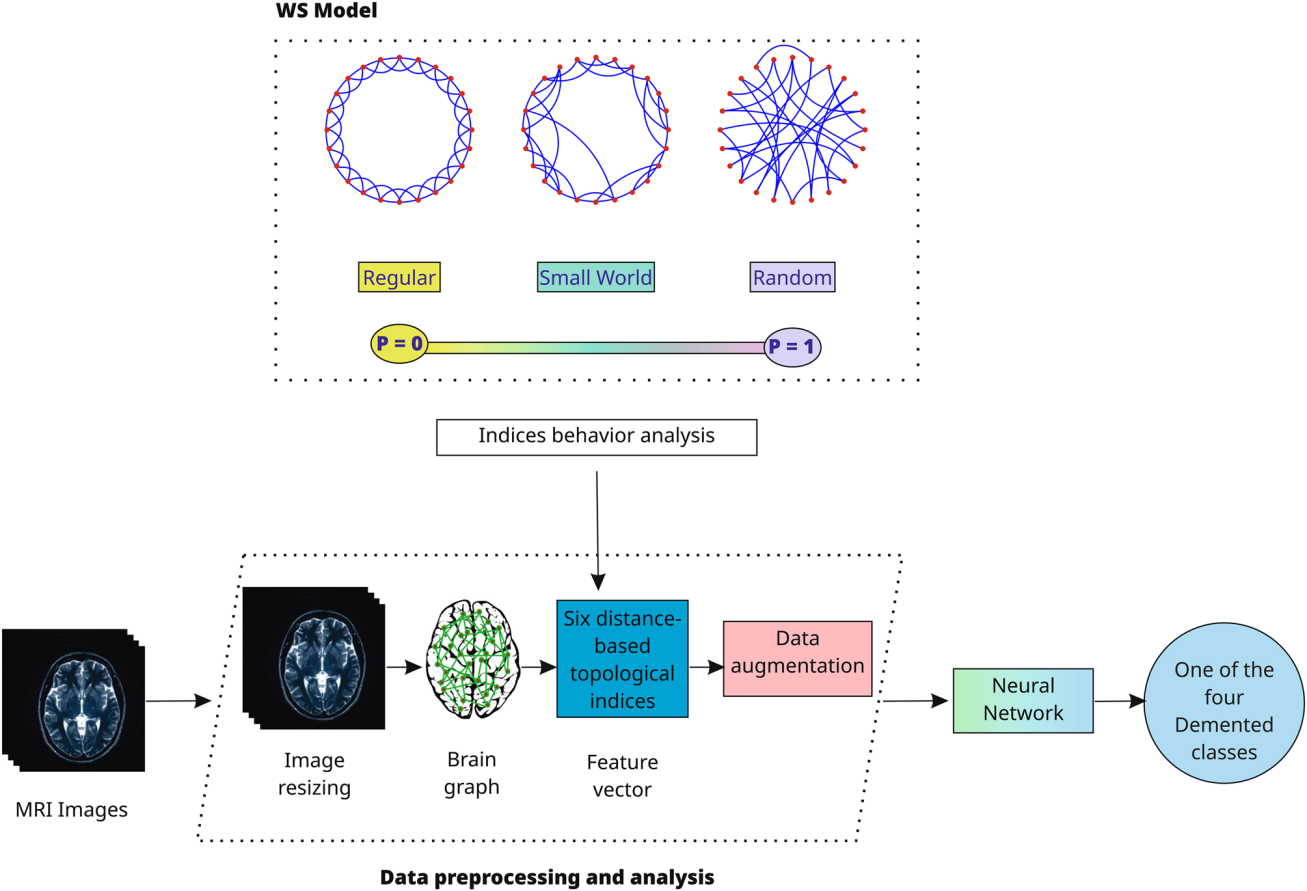
The brain graph analysis pipeline from MRI acquisition to classification. The proposed framework begins with the construction of brain graphs from MRI images, followed by the computation of distance-based topological indices and normalization using the WS model. These graph-based features are then used to train machine learning models, culminating in the classification of Alzheimer’s disease stages.

## Methodology

### Proposed model’s workflow

The proposed model follows a systematic workflow to extract and analyze topological features from brain networks constructed using MRI data (Fig. 1). The process begins by converting brain MRIs into graphs using the BDM process. This approach uses the distances between pixel intensities to calculate useful statistical values and image features for pattern recognition and classification.

Since brain networks are known to display small-world properties, the constructed brain graphs were modeled using the WS model to better capture their topological organization. Although this model has been widely used to simulate various real-world networks, its application for modeling brain graphs represents a novel aspect of this study. This approach offers potential for uncovering meaningful insights into the structure and function of brain networks. The resulting model properties were then interpreted using distance-based topological indices, which were employed to assess the connectivity and efficiency of these brain networks.

After extracting the topological indices, machine learning model was applied to classify the four stages of Alzheimer’s Disease. The model used the computed topological indices from MRI-derived brain graphs as input features. As only numerical values describing the brain network structures were utilized, no personal patient information was exposed, ensuring compliance with the privacy requirements of federated learning frameworks. This classification approach achieved a precision of approximately 89.45%.

### Watts and Strogatz model

The WS model^27^ is a widely used framework for generating random graphs that exhibit the characteristics of small-world networks. These networks are known for their short average path lengths and high clustering coefficients. The model begins by creating a ring lattice^30^ consisting of *v* vertices, labelled from 0 to $v-1$ in a clockwise sequence. Each vertex is initially connected to its $\delta$ nearest neighbor through undirected edges. Starting from vertex 0, the edge connected to the nearest neighbor [$i+1 \ (\textrm{mod} \ v)$] is selected for each vertex *i*. Then, with a probability *p*, each edge is either retained or rewired to a randomly chosen vertex on the ring lattice, avoiding duplicate connections and self-loops. This process continues sequentially around the ring, first considering the nearest neighbors, then second-nearest neighbor, and so on, applying the rewiring probability *p* to each edge. By adjusting the value of *p* from 0 to 1, the model transitions between a regular lattice and a random network while attaining the small-world properties in between 0 and 1. In this study, we analyze the topological properties of these generated networks using distance-based topological indices.

### Topological indices

The topological indices of a graph are numerical values that describe the characteristics of the graph’s structure and connectivity. These indices serve as quantitative descriptors that capture various properties such as distances, branching, and overall network organization, making them valuable tools for analyzing complex systems like brain networks. In a finite, undirected graph $H$, let $\mathcal {N}(H)$ represent the set of nodes and $\mathcal {B}(H)$ represent the set of edges. The shortest path distance between any two vertices $r$ and $s$ in $H$ is given by $\rho (r, s)$, measured by the minimum number of edges connecting them.

For any edge $e = rs$ in $H$, the set of vertices closer to $r$ than to $s$ is defined as:

$$\Lambda _r(e) = \{t \in \mathcal {N}(H) \mid \rho (r, t) < \rho (s, t)\}$$

Similarly, the set of vertices closer to $r$ than to $s$ is given by:

$$\Lambda _s(e) = \{t \in \mathcal {N}(H) \mid \rho (s, t) < \rho (r, t)\}$$

The cardinality of these sets are denoted as $|\Lambda _p(r)| = \alpha _r$ and $|\Lambda _q(s)| = \alpha _s$. Based on these values, the Szeged index of the graph H is calculated as:

$$Sz(H) = \sum _{rs \in \mathcal {B}(H)} \alpha _r \cdot \alpha _s$$

The Szeged index serves as an extension of the Wiener number for cyclic structures derived from distance matrices. It has applications not only in chemical graph theory but also in various other domains^31^. Ilić^32^ further refined the concept by focusing only on edges $e = rs$ where, $\alpha _r = \alpha _s$. A graph in which all edges satisfy this property is termed distance-balanced. Such graphs hold significance in graph theory and its applications. Notably, this article demonstrates that the WS model at $p = 0$ represents an example of a distance-balanced graph. In addition to the Szeged index, several other distance-based topological indices^33^ have been used to characterize the structural and connectivity properties of complex networks. These indices capture various aspects of node centrality, peripheral balance, and path-based properties within a graph. The expressions for the distance-based topological indices adopted in this study are as follows:

- **Graovac–Ghorbani (ABC) index:**
  $$ABC(H) = \sum _{rs \in \mathcal {B}(H)} \sqrt{\frac{\alpha _r + \alpha _s - 2}{\alpha _r \cdot \alpha _s}}$$
- **PI index:**
  $$PI(H) = \sum _{rs \in \mathcal {B}(H)} (\alpha _r + \alpha _s)$$
- **Mostar index:**
  $$MO(H) = \sum _{rs \in \mathcal {B}(H)} \left| \alpha _r - \alpha _s \right|$$
- **Normalized Graovac–Ghorbani (NGG) index:**
  $$NGG(H) = \sum _{rs \in \mathcal {B}(H)} \frac{1}{\sqrt{\alpha _r \cdot \alpha _s}}$$
- **Wiener index:**
  $$W(H) = \sum _{r,s \in \mathcal {N}(H)} \rho (r, s)$$

After defining these indices, brain graphs were extracted from MRI data for further analysis.

### Data preprocessing

#### Dataset

The dataset utilized in this study was obtained from the publicly available Kaggle database (https://www.kaggle.com/datasets/tourist55/alzheimers-dataset-4-class-of-images), which provides a large collection of structural brain MRI scans. It consists of approximately 5,000 T1-weighted MRI images, organized into two primary folders: *Training* and *Testing*. These images are classified into four categories based on the severity of Alzheimer’s Disease (AD): Non-Demented, Very Mild Demented, Mild Demented, and Moderate Demented. Each category represents a different stage of disease progression, allowing the model to learn and classify brain network patterns associated with varying levels of cognitive impairment.

The dataset complies with the Open Database License (ODbL), ensuring that it can be freely accessed and redistributed with proper attribution. All images in the dataset are provided in JPEG format, pre-processed to a uniform size of $176 \times 208$ pixels. No personal or sensitive patient information is included, making the dataset suitable for research applications involving privacy-preserving frameworks, such as federated learning.

#### The construction of brain graphs

**Fig. 2:**
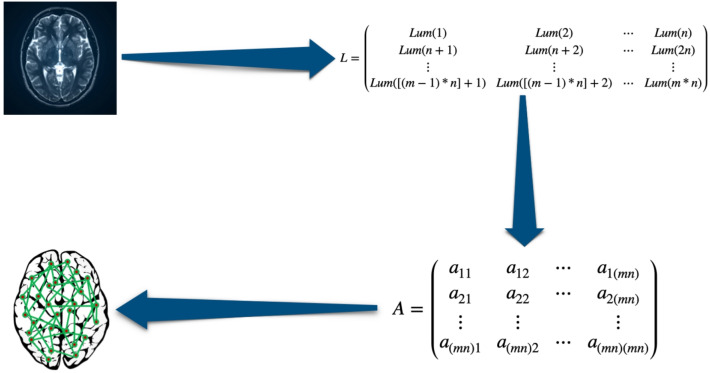
Pipeline for brain graph generation: The MRI image is converted into a luminance matrix (L), which is then transformed into an adjacency matrix (A) representing the brain network.

The obtained MRI data has to be converted into a graph representation to analyze the brain network. The graph construction process involves creating an adjacency matrix based on the pixel brightness values of the MRI images (Fig. 2). Each pixel in the image is treated as a node in the graph, and edges are established between the nodes based on the similarity of their brightness values. The strength of these edges is determined by the brightness difference between connected pixels, allowing for a meaningful representation of the brain’s structural connectivity^25^. Here are the steps to create the graph:

- Construction of Brightness Difference Matrix (BDM)
- Normalization of BDM
- Adjacency Matrix Generation via Thresholding

#### Construction of brightness difference matrix (BDM)

To construct the matrix, we first separate the brightness (or luminance) indicators of each pixel. Given $mn$ pixels, we obtain an $mn \times mn$ matrix, where each element is computed using the formula:

$$L(p, q) = |Lum(p) - Lum(q)|$$

Here, $L(p, q)$ denotes the luminance difference between pixels $p$ and $q$, and $Lum(p)$ is the brightness value of pixel $p$.

#### Normalization of BDM

The computed BDM values are then normalized to a standard range between 0 and 1 to ensure consistency in edge weight interpretation. This is performed using the following equation:

$$L_n(p, q) = \frac{L(p, q) - \min (L)}{\max (L) - \min (L)}$$

where $L_n(p, q)$ denotes the normalized luminance difference between pixels p and q, and $\min (L)$ and $\max (L)$ represent the minimum and maximum values of the BDM, respectively.

#### Adjacency matrix generation via thresholding

Once the BDM is normalized, an adjacency matrix is generated to represent the brain network’s connectivity. In this matrix, pairs of pixels with similar brightness values are considered neighbors, while those with significant differences are not connected. This step transforms image-based information into a network structure suitable for graph-based analysis. The adjacency matrix $A$ is constructed according to the following rule:

1 $$\begin{aligned} A(p, q) = {\left\{ \begin{array}{ll} 1 & \text {if } 1 - L_n(p, q) \ge \tau \\ 0 & \text {otherwise} \end{array}\right. } \end{aligned}$$

Here, $\tau$ is a predefined threshold that controls the connectivity density of the resulting graph by determining which pixel pairs are connected. A higher value of $\tau$ results in a sparser graph, while a lower value increases connectivity.

In the adjacency matrix A(p, q), each entry indicates whether a connection exists between two pixels based on the threshold value. Connections from a node to itself (self-loops) are removed to preserve meaningful network structure. This graph is then used to calculate distance-based topological indices, which describe different aspects of the network’s structure and connectivity, providing numerical insights into the brain’s organization.

### Exploratory data analysis of indices

The six distance-based topological indices were computed from the adjacency matrices of the brain graphs. These indices act as numerical descriptors of the brain network’s topology, capturing important features such as clustering, path length, and neighborhood connectivity. To ensure comparability, the indices were normalized using the WS model, placing them on a consistent scale suitable for machine learning applications.

**Fig. 3:**
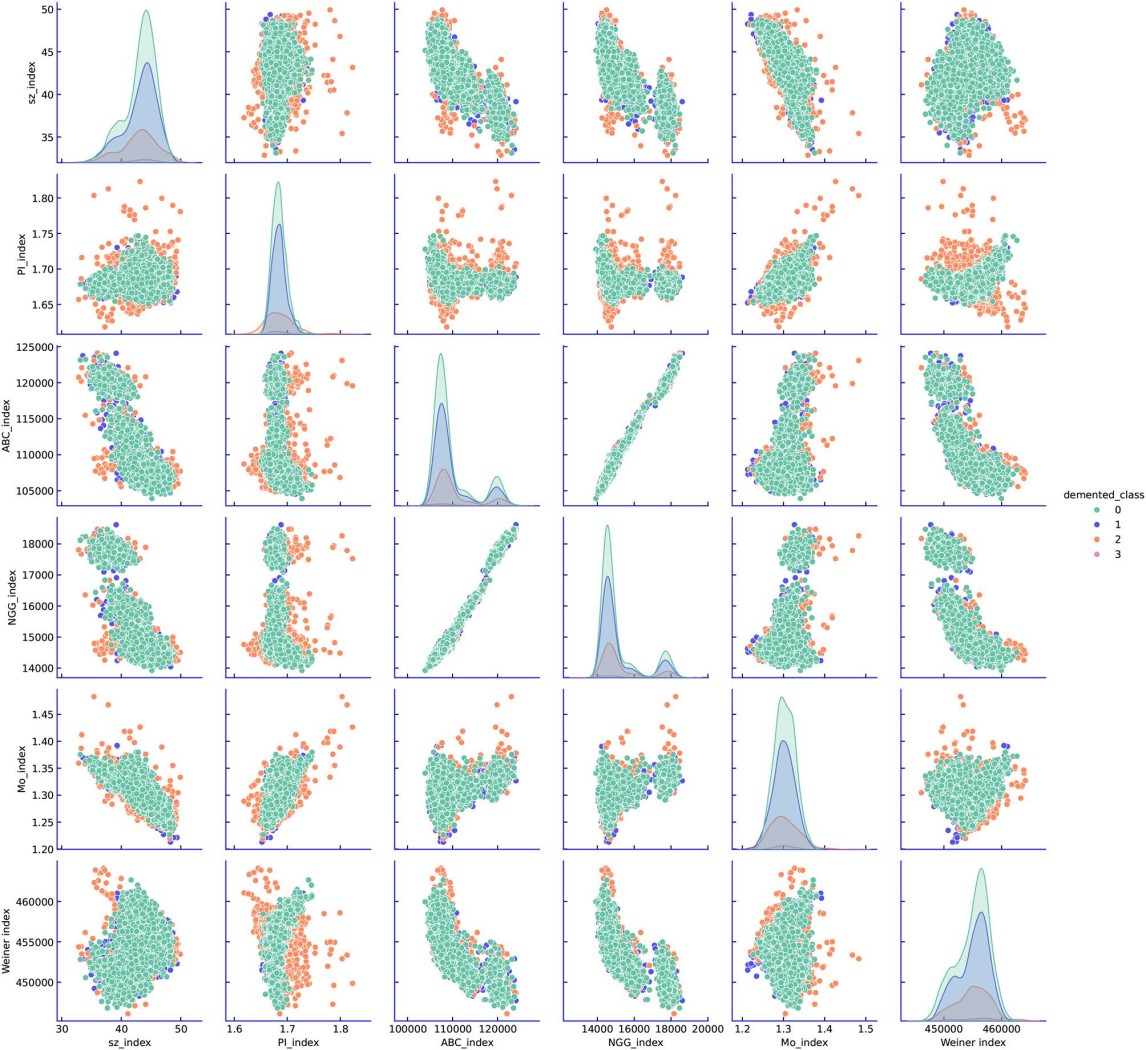
Data visualisation. Classes 0, 1, 2, and 3 represent Non-Demented, Very Mild Demented, Mild Demented, and Moderate Demented, respectively. The pair plot indices, displayed in the Seaborn plot, use the Demented class as the reference point for comparison across six indices.

An detailed data analysis was then conducted to examine the distribution of these normalized indices across different stages of AD. Pair plots were generated to visualize the relationships among the six indices, with particular focus on identifying patterns associated with the demented class. This visualization provided preliminary insights into how the brain’s connectivity properties differ with disease progression and helped assess the separability of classes based on the topological indices (Fig. 3).

### Handling class imbalance

The dataset exhibited an imbalance between dementia classes, which could negatively impact classification model performance. To address this, the Synthetic Minority Over-sampling Technique (SMOTE) was applied to the training data. SMOTE generates synthetic samples for the minority classes by interpolating between existing samples in the feature space. This balanced the class distribution within the training set while leaving the test data unchanged, ensuring a fair evaluation of model.

### Data splitting and cross-validation strategy

The dataset was partitioned into training and testing sets using an 80-20 split. To further evaluate the model’s robustness, K-fold cross-validation was applied to the training data, with stratification to maintain class proportions in each fold. This approach helped assess model performance on unseen data and minimized the risk of overfitting.

Before proceeding to the classification phase, a detailed analysis on the path-based indices was conducted to understand their behavior in the WS model. For clarity, the key parameters used throughout this study are as follows: The parameter *v* represents the number of vertices (nodes) in a network, while $\delta$ indicates the number of nearest neighbors each vertex initially connected in the WS model. The rewiring probability *p* determines the likelihood of replacing a regular connection with a random one, controlling the network’s small-world characteristics. The threshold $\tau$ is used to convert the normalized BDM into an adjacency matrix. The shortest path distance between vertices is denoted by $\rho (r, s)$, and $\alpha _r$, $\alpha _s$ represent the number of vertices closer to a given vertex for a particular edge.

## Results and discussion

### The Behaviour of Path-based indices in the WS model

In network analysis, path-based indices are a helpful tool to measure the distance between paths and identify clustering levels. Our theorem 1 introduces a generalized method to calculate neighborhood values in the WS model, regardless of the values of *v* and $\delta$, given that probability p is set to 0. The theorem is proved, based on the assumption that $\delta < \frac{v}{2}$, since any two vertex at most have a distance of 2. But this theorem works in that case also but with a slight modification.

#### Theorem 1

*Let* $H$ *be a* $\delta$*-regular WS model with* *v* *vertices. If* $v - 1 \equiv \gamma \ (\text {mod} \ \delta )$ *and* $C = \Big \lfloor \frac{v - 1}{\delta } \Big \rfloor$,* then for any edge* *rs*, *where* $r = i$ *and* $s = i + u \ (\text {mod} \ v)$ *with* $u \le \frac{\delta }{2},$

$$\alpha _u = \alpha _z = {\left\{ \begin{array}{ll} u \cdot (C - 1) + \gamma & \text {if } \gamma < u \\ u \cdot C + 1 & \text {otherwise} \end{array}\right. }$$

### Proof

Let *G* be a $\delta$-regular WS model graph. The proof is first established for vertex $i = 0$ and *u* ranging from 1 to $\frac{\delta }{2}$, and then generalized for any vertex *i* in the model.

Assuming $i = 0$ and *u* ranging from 1 to $\frac{\delta }{2}$. If $u = 1$, the edge between vertices 0 and 1 is considered. Vertices 2 to $\frac{\delta }{2}$ are at a distance of one from both vertices 0 and 1. However, only vertex 1 has an edge on the right half of the WS model when it comes to vertex $\frac{\delta }{2} + 1$. Similarly, the vertex $[2 \cdot \Big (\frac{\delta }{2}\Big ) + 1]$ has a distance of two on the right half from vertex 1. Repeating this process until the left side reaches faster than the right (denote this point as $z_1$):

$$\Lambda _1(01) = \left\{ 1, \frac{\delta }{2} + 1, 2 \cdot \frac{\delta }{2} + 1, \dots , z_1 \cdot \frac{\delta }{2} + 1 \right\}$$

$$\alpha _z = |\Lambda _1(01) |= z_1 + 1$$

Similarly, for the left side of vertex 0:

$$\Lambda _0(01) = \left\{ 0, \ v - \frac{\delta }{2}, \ v - 2 \cdot \frac{\delta }{2}, \dots , \ v - z_0 \cdot \frac{\delta }{2}\right\}$$

Finding the maximum $z_1$ such that $z_1 * \frac{\delta }{2} + 1 \ge v - z_1 * \frac{\delta }{2}$ will not happen:

$$z_1 \cdot \frac{\delta }{2} + 1 < v - z_1 \cdot \frac{\delta }{2}$$

$$z_1 < \frac{v - 1}{\delta }$$

Since $z_1$ should be maximized, we have:

$$z_1 = {\left\{ \begin{array}{ll} \Big \lfloor \frac{v - 1}{\delta } \Big \rfloor - 1 = C - 1 & \text {if } \frac{v - 1}{\delta } \text { is an integer} \\ \\ \Big \lfloor \frac{v - 1}{\delta } \Big \rfloor = C & \text {otherwise} \end{array}\right. }$$

$$\implies \alpha _1 = {\left\{ \begin{array}{ll} C & \text {if } \frac{v - 1}{\delta } \text { is an integer} \\ C + 1 & \text {otherwise} \end{array}\right. }$$

If $v - 1 \equiv 0 \ (\text {mod} \ \delta )$, then $\gamma = 0$ and $u = 1$, giving:

$$\alpha _1 = {\left\{ \begin{array}{ll} u \cdot (C - 1) + \gamma & \text {if } \gamma < u \\ u \cdot C + 1 & \text {otherwise} \end{array}\right. }$$

Applying the same argument to $z_0$:

$$\alpha _0 = {\left\{ \begin{array}{ll} u \cdot (C - 1) + \gamma & \text {if } \gamma < u \\ u \cdot C + 1 & \text {otherwise} \end{array}\right. }$$

Now consider the edge 0*u*, where $u \le \frac{\delta }{2}$. The vertices adjacent to *u* but not to 0 are $\left( \frac{\delta }{2}\right) + 1, \ \left( \frac{\delta }{2}\right) + 2, \ \cdots , \ \left( \frac{\delta }{2}\right) + u$ and

$$\Lambda _u(0u) = \left\{ 1, \ \frac{\delta }{2} + 1, \ \frac{\delta }{2} + 2, \ \dots , \frac{\delta }{2} + u, \ 2 \cdot \frac{\delta }{2} + 1, \ \dots , \ z_u \cdot \frac{\delta }{2} + \gamma _1 \right\} ,$$

where $$1 \le \gamma _1 \le u$$.

Applying the similar approach to 01:

**Case 1:** If $\gamma _1 = u$ and $v - 1 \not \equiv 0 \ (mod \ \delta )$. $z_r = C$ (by 2), and

$$\begin{aligned} \alpha _u = u \cdot C + 1 \end{aligned}$$

**Case 2:** If $\gamma _1 = u$ and $v - 1 \equiv 0 \ (mod \ \delta )$. $z_r = C - 1$ (by 2), and

$$\begin{aligned} \alpha _u = u \cdot (C-1) + 1 \end{aligned}$$

**Case 3:** If $$\gamma _1 < r$$,

$$\begin{aligned} \iff z_r \cdot \left( \frac{\delta }{2} \right) + 1 + \gamma _1 \ \ge \ v - z_r \cdot \left( \frac{\delta }{2} \right) \ \text {and} \ z_r \cdot \left( \frac{\delta }{2} \right) + 1 + \gamma _1 - 1 \ > \ v - z_r \cdot \left( \frac{\delta }{2} \right) \ \end{aligned}$$

$$\begin{aligned} \iff z_r \cdot k + \gamma _1 + 1 \ \ge \ v \ \text {and} \ z_r \cdot k + \gamma _1 \ < \ v \end{aligned}$$

$$\begin{aligned} \iff z_r \cdot k + \gamma _1 + 1 \ \ge \ v \ > \ z_r \cdot k + \gamma _1 \end{aligned}$$

$$\begin{aligned} \iff z_r \cdot k + \gamma _1 + 1 \ = \ v \end{aligned}$$

$$\begin{aligned} \implies \gamma _1 \ \equiv \ v - 1\ (mod \ \delta ) \end{aligned}$$

if $$\gamma _1 \ \equiv \ v - 1\ (mod \ \delta )$$ then,

$$\begin{aligned} z_r \ = \ \frac{n - 1 - \gamma _1}{\delta } \implies z_r \cdot k + \gamma _1 + 1 \ = \ v \end{aligned}$$

Therefore,

$$\begin{aligned} \gamma \ \equiv \ v - 1\ (mod \ \delta ) \iff u \cdot (C - 1) + \gamma _1 \end{aligned}$$

Concluding all cases, if $\gamma \ \equiv \ v - 1\ (mod \ \delta )$

$$\alpha _u = {\left\{ \begin{array}{ll} u \cdot (C - 1) & \text {if } \gamma = 0 \\ u \cdot (C - 1) + \gamma & \text {if } 1 \le \gamma < u \\ u \cdot C + 1 & \text {otherwise} \end{array}\right. }$$

$$\alpha _u = {\left\{ \begin{array}{ll} u \cdot (C - 1) + \gamma & \text {if } 0 \le \gamma < u \\ u \cdot C + 1 & \text {otherwise} \end{array}\right. }$$

Similarly, the theorem can be proved for any vertex i. Hence, the results hold for all the vertices of the graph.

**Fig. 4:**
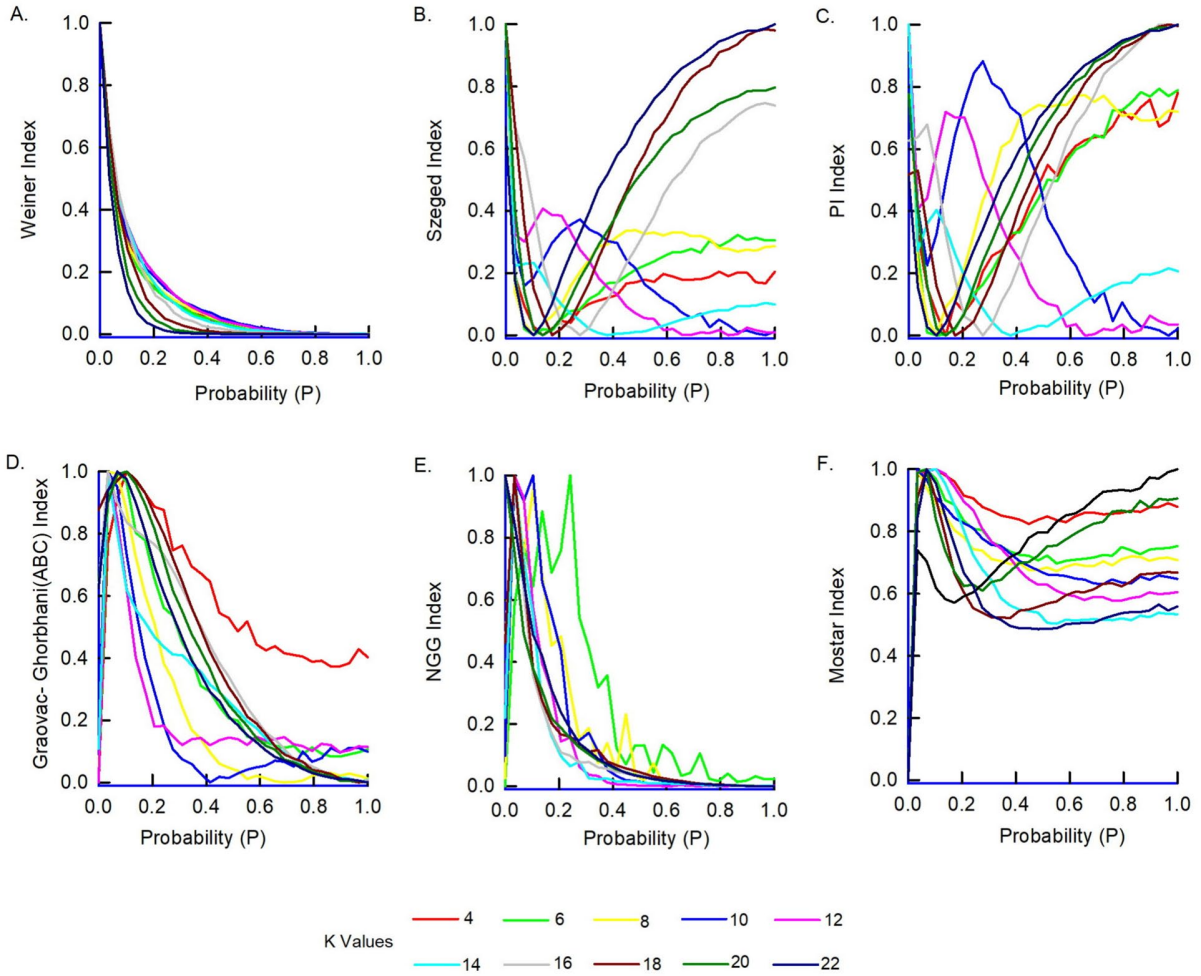
Path-based indices in WS model. Six topological indices: (**A**) Weiner Index, (**B**) Szeged Index, (**C**) PI Index, (**D**) Graovac-Ghorbhani Index, (**E**) NGG Index, (**F**) Mostar Index, which are associated with the normalized graph of the WS model G. This graph comprises 50 vertices, and the parameter k varies from 4 to 22.

We also computed all the indices of the WS model for $v = 50$ and $\delta$ values ranging from 4 to 22, across all probabilities p. To obtain these results, we generated 2000 graphs for each probability p and $\delta$ value, using algorithms, calculated the average value of all indices, and presented the findings in Fig. 4.

A distinct global minimum or maximum is observed for all indices within the probability range of p = 0.2 to 0.3 across $\delta$ values ranging from $4 \text { to } 22$ for all the indices. The specific occurrence of a minimum or maximum depends on how each index accounts for neighborhood values. This pattern suggests an inverse relationship between neighborhood connectivity and small-worldness. When $\delta$ exceeds *v*/2 (i.e., 25 in this study case), the WS graphs display a uniform increase or decrease in index values. This occurs because at $p = 0$, the distance between any two vertices is nearly 2 for $\delta > v/2$, creating a highly clustered structure. As the rewiring probability increases, the small-world property diminishes, and the indices become less sensitive to further structural changes.

These results support our hypothesis that distance-based topological indices are sensitive to changes in network structure, especially in small-world networks. The occurrence of distinct minima or maxima for various indices around the probability range of p = 0.2–0.3 shows that these indices can effectively capture how the network’s structure changes. This finding strengthens our assumption that similar indices, when applied to brain graphs from MRI images, could help detect connectivity disruptions linked to Alzheimer’s disease. Although the WS model and various graph indices have been extensively used in previous studies, the novelty of this work lies in performing a systematic ablation study by varying neighborhood sizes $\delta$ and rewiring probabilities *p*. This not only confirms the established behaviors in synthetic WS networks but also quantifies the sensitivity ranges of different graph indices. Additionally, these observations are directly applied to brain-derived connectivity networks, providing a unique perspective on the sensitivity of graph indices in the context of AD. To our knowledge, such a combined evaluation on both simulated and real brain networks has not been previously reported.

**Table 1:** Comparision of mean and standard deviation of clustering coefficient (C) and average path length (L) for brain graphs and WS graphs across AD stages.

Stage	Clustering coefficient (C)		Average path length (L)	
	Brain graphs	WS graphs	Brain graphs	WS graphs
Very mild demented	0.8006 ± 0.0024	0.7258 ± 0.0010	1.4103 ± 0.0051	1.4118 ± 0.0054
Mild demented	0.8002 ± 0.0015	0.7257 ± 0.0009	1.4101 ± 0.0047	1.4116 ± 0.0046
Moderate demented	0.7997 ± 0.0011	0.7257 ± 0.0007	1.4098 ± 0.0027	1.4111 ± 0.0031
Non demented	0.8035 ± 0.0023	0.7259 ± 0.0011	1.4118 ± 0.0041	1.4126 ± 0.0037

All values are expressed as Mean ± SD.

### Topological indices in brain network analysis

To support the rationale for our graph-theoretic approach, it is essential to recognize how these properties are exhibited in the structural and functional networks of the human brain. Specifically, research that used non-invasive diffusion imaging to study the brain’s large-scale structural networks, the nodes organized into structural communities or modules. This finding suggests a tendency towards modularity in the brain, which is accompanied by a high capacity for global information flow, as evidenced by its efficiency and short path length. These features are characteristic of small-world networks, and have been consistently observed in studies of whole-brain structural networks.

Two key characteristics of these networks are functional integration (measured by global efficiency) and functional segregation (measured by local clustering). The small-world nature of brain networks supports both properties. Such a structure can be replicated using the WS model by selecting appropriate rewiring probabilities^34^. These types of brain network structures can be analyzed using topological indices. In this study, path-based indices are used to capture the brain’s functional integration, while clustering-based indices reflect functional segregation, as they quantify the local neighborhood connectivity patterns in networks constructed from MRI images.

To further investigate the small-world characteristics of brain networks in Alzheimer’s disease, we compared the clustering coefficient and average path length of brain graphs with those of equivalent WS model graphs across different disease stages. The results, summarized in Table 1, demonstrate that while both brain and WS graphs exhibit small-world properties, brain graphs consistently showed higher clustering coefficients across all AD stages. This indicates stronger local connectivity in brain networks, a hallmark of small-worldness in biological systems. The average path lengths remained comparable between the two, reinforcing the presence of efficient global integration within the brain networks. These observations support the appropriateness of applying the WS model for normalizing brain graphs and highlight meaningful topological alterations associated with disease progression.

With the behavior of the path-based and clustering-based indices characterized within the WS model framework, their utility was next assessed in classifying MRI-derived brain networks. The set of normalized topological indices computed from patient brain graphs served as input features for this task. Four classification models were developed to evaluate their effectiveness in distinguishing between dementia categories: a Support Vector Machine (SVM), a Neural Network with two hidden layers, a Decision Tree, and Logistic Regression.

### Statistical analysis

A variety of evaluation methods were used to assess each model’s performance. For the SVM model, we employed the Radial Basis Function (RBF) kernel, a widely used kernelization technique similar in form to the Gaussian distribution. The RBF kernel computes the similarity between two data points based on their Euclidean distance and is mathematically expressed as:

$$\begin{aligned} K(X, X') = exp (-\frac{\Vert {X - X'}\Vert ^2}{2 \sigma ^2}) \end{aligned}$$

Here, $\sigma$ s the kernel width (variance) and a tunable hyperparameter, typically selected based on the specific data characteristics and analysis requirements. In this study, $\sigma$ was set to 1.

For evaluating the two-hidden-layered neural network, we used Categorical Cross-Entropy (CCE) as the loss function, as the dataset involves multi-class labels. In this model, the output is represented as a one-hot encoded vector y, where each element is set to 1 for the correct class and 0 for all others. The CCE loss function measures the dissimilarity between the true and predicted class probabilities and is defined as:

$$\begin{aligned} J(y, y') = - \sum (y_i * log(y'_i)) \end{aligned}$$

Here, $y_i$ represents the example’s real probability of belonging to class *i*, while $y'_i$ represents the example’s anticipated likelihood of belonging to class *i*.

For the Decision Tree and Logistic Regression classifiers, the accuracy score is used to evaluate model performance. This score is computed as the ratio of the number of correct predictions to the total number of input samples.

**Fig. 5:**
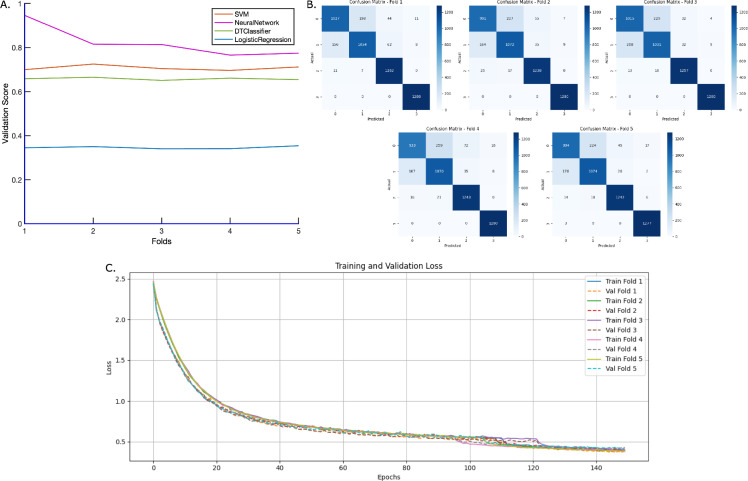
Comparison of model performance metrics and loss values across different training configurations for the classification of four classes. (**A**) Accuracy comparison of various models, including Decision Tree, Logistic Regression, SVM, and Neural Network. Among these, the Neural Network model achieved notably higher accuracy compared to the others. (**B**) Confusion matrices obtained from five-fold cross-validation of the optimized Neural Network model. (**C**) Validation loss curves across 150 epochs for each fold during five-fold cross-validation of the optimized Neural Network model.

### Model construction and comparisons

After evaluating each model using appropriate statistical methods, we proceeded to compare their classification performance through K-fold cross-validation. Given the clinical importance of reliable classification in AD, accuracy was emphasized as the primary evaluation metric, alongside other supporting measures.

The primary objective of this study was to develop a learning model capable of accurately distinguishing between individuals with Alzheimer’s Disease (AD) and healthy controls based on graph-derived topological features. To evaluate classification performance, four models were trained and validated: Decision Tree, Logistic Regression, Support Vector Machine (SVM), and Neural Network (Fig. 5A). Both the Decision Tree and Logistic Regression models demonstrated limited performance, despite hyperparameter tuning. The Decision Tree model is prone to overfitting, particularly with high-dimensional numerical datasets like graph-based indices, and often struggles to capture complex, non-linear relationships inherent in brain connectivity patterns. Logistic Regression, being a linear classifier, is inherently limited in separating non-linearly distributed data, making it unsuitable for datasets where disease-related variations manifest through intricate network structures. The SVM classifier achieved moderately better accuracy of approximately 70%, owing to its ability to handle non-linear relationships through kernel functions. However, its performance was limited by the presence of overlapping distributions in the topological feature space across the four disease stages, which complicates margin-based separation. In contrast, The Neural Network with two hidden layers outperformed the other classifiers, achieving an accuracy of 85%, and was thus selected for further analysis. To improve its performance further, the model was expanded to four hidden layers with multiple dropout layers to reduce overfitting, and the number of training epochs was increased to 150.

To assess the stability and generalization ability of this improved model, a five-fold cross-validation was performed. The confusion matrices obtained from each fold (Fig. 5B) provide a detailed breakdown of the correctly and incorrectly classified instances for both AD and healthy control groups. These matrices not only show the classification accuracy but also reveal how consistently the model performed for each disease stage across different validation subsets.

In addition to classification accuracy, the validation loss values were recorded over 150 epochs for each fold to monitor the training progress and model convergence. The validation loss curves (Fig. 5C) indicate a steady and consistent decline across all folds, demonstrating stable learning behavior and reduced overfitting risk. The consistent convergence patterns further confirm the reliability and robustness of the optimized Neural Network model in accurately classifying individuals into the four disease stages and the healthy control group.

**Table 2:** Fold-wise classification performance metrics obtained from five-fold cross-validation of the optimized Neural Network model.

Fold	Accuracy	Precision	Recall	F1-score	AUC
1	0.9029	0.9013	0.9029	0.9016	0.9821
2	0.8947	0.8936	0.8947	0.8937	0.9788
3	0.8951	0.8936	0.8951	0.8942	0.9793
4	0.8840	0.8827	0.8840	0.8821	0.9766
5	0.8959	0.8947	0.8959	0.8949	0.9772
Mean	0.8945	0.8932	0.8945	0.8933	0.9788

The table reports accuracy, precision, recall, F1-score, and AUC for each fold.

To further validate the model’s performance, multiple evaluation metrics were computed for each fold during cross-validation, including accuracy, precision, recall, F1-score, and the area under the receiver operating characteristic curve AUC. The fold-wise results for these metrics are summarized in Table 2.

The model demonstrated consistently high performance across all folds, with accuracy values ranging from 88.40% to 90.29%, and an average accuracy of 89.45%. Precision and recall values remained closely matched, with averages of 89.32% and 89.45%, respectively, reflecting balanced sensitivity and positive predictive value. Similarly, the F1-score averaged 89.33%, indicating reliable overall classification effectiveness. Importantly, the model achieved consistently high AUC values across all folds, ranging from 0.9766 to 0.9821, with an average of 0.9788, confirming excellent discriminative capability. The evaluation metrics across multiple folds further validates the robustness, and potential clinical utility of the proposed Neural Network framework for AD stage classification.

**Table 3:** The table presents a detailed analysis of the proposed model, highlighting its importance and potential benefits.

Literature	Method	Federative learning	Accuracy
Ebrahimi^13^ (2021)	CNN	No	91 %
Orouskhani^12^ (2022)	Deep triplet network	No	99%
Kavitha^3^ (2022)	Four different ML Models	No	83.4%
Dogan^20^ (2022)	Graph approach PBP algorithm	No	92.5%
Shrivastava^9^ (2023)	Four different ML Models	No	80%
Nimeshika^35^ (2024)	Split-FL + conditional GAN	Yes	83.5
Proposed model	Graphical approach topological indices	Yes (Uses graph characteristics)	89.45%

Table 3 presents a comparative overview of recent AD classification models, highlighting their methodological approaches, reported accuracies, and support for federative learning frameworks. Conventional deep learning models, such as the CNN proposed in 2021^13^ and the deep triplet network introduced in 2022^12^, achieved high classification accuracies of 91% and 99%, respectively. However, these models primarily function as black-box systems, offering limited interpretability and no support for federative learning, which limits their suitability for collaborative, privacy-sensitive clinical applications.

Other approaches employing multiple traditional machine learning models^3,9^ reported moderate accuracies ranging from 80% to 83.4%, but similarly lacked integrated privacy-preserving frameworks or mechanisms for explaining model predictions. A graph-based Pattern-Based Prediction (PBP) algorithm developed in 2022^20^ improved interpretability through graph features and achieved 92.5% accuracy, yet did not incorporate federative learning capabilities. More recently, the Split-FL with GAN-based augmentation model^35^ demonstrated 83.5% accuracy within a federative learning environment, addressing privacy concerns but without offering transparent, interpretable biomarkers for individualized disease staging.

In contrast, the proposed model in this study achieved a competitive accuracy of 89.45% while uniquely combining federative learning with a graph-based framework centered on distance-based topological indices extracted from brain networks. These topological indices, including measures such as average path length, clustering coefficient, and eccentricity, offer quantifiable, interpretable biomarkers that reflect structural alterations in brain connectivity associated with AD. By modeling patient-specific brain graphs and analyzing their topological properties, the framework provides a biologically meaningful representation of disease progression, a capability notably absent from prior models emphasizing prediction accuracy alone.

Furthermore, the proposed model supports federative learning, enabling collaborative model training across multiple healthcare centers while preserving patient data privacy. This dual emphasis on interpretability and data security positions the proposed framework as a practical and scalable solution for real-world, multi-center AD classification and staging. The inclusion of federative learning ensures compliance with data governance standards, while the topological indices contribute critical biological insights, making the predictions both explainable and clinically actionable.

Additionally, the framework supports individualized stage-wise detection of AD, an essential aspect for clinical decision-making, as it enables physicians to monitor disease progression patterns and tailor patient management strategies accordingly. The model’s training and evaluation processes were conducted using open-source libraries, including scikit-learn and TensorFlow within a Python environment, promoting reproducibility and facilitating future extensions.

Overall, this study moves beyond conventional emphasis on accuracy alone by integrating interpretable, topologically derived biomarkers within a privacy-preserving framework. The findings suggest that topological indices derived from MRI-based brain graphs not only enhance classification accuracy but also serve as biologically meaningful markers for staging and tracking the progression of AD.

### Limitations and future work

Although the findings of this study are encouraging, several limitations should be acknowledged. The dataset used was relatively small and limited to a single-center source, which may restrict the broader applicability of the model to diverse clinical populations. To improve the clinical relevance and robustness of the proposed framework, future work will focus on validating this approach using larger, multi-center datasets with a wider range of demographic and clinical characteristics. Additionally, the current analysis was restricted to structural MRI data. Incorporating additional neuroimaging modalities such as functional MRI (fMRI) and diffusion tensor imaging (DTI) could provide complementary perspectives on both structural and functional brain network alterations associated with Alzheimer’s disease. This multimodal integration has the potential to enhance the sensitivity of graph-based topological indices in detecting subtle disease-related changes.

Beyond neuroimaging, this graph-based framework can also be extended to medical signal analysis tasks, such as EEG and ECG, where connectivity patterns between channels or signal segments are commonly represented as networks. Since EEG and ECG connectivity networks function similarly to fMRI-derived brain graphs in structure and analysis, the proposed topological index-based model could be directly adapted for studying signal-based network disruptions in neurological and cardiac disorders. Further work will also investigate integrating additional graph-theoretic measures and combining these with imaging biomarkers to improve diagnostic accuracy and clinical relevance. Implementing this model within federated learning environments remains a future priority to enable secure, collaborative clinical studies.

## Conclusion

This study proposed a graph-based framework for analyzing brain network disruptions in Alzheimer’s disease using distance-based topological indices derived from MRI images. The WS small-world network model was applied to study the small-world properties of patient brain networks and to normalize these indices, thereby improving their interpretability and enabling fair comparisons across patient brain graphs. The comparative analysis demonstrated that brain graphs consistently exhibited higher clustering coefficients than equivalent WS networks, while maintaining comparable average path lengths, confirming the presence of small-world characteristics in both healthy and diseased brains. Machine learning models trained on these graph-based features, particularly a refined Neural Network model, achieved a classification accuracy of 89.45%, confirming the utility of these indices for effective disease staging. The proposed framework emphasizes interpretable, graph-theoretic features derived from brain network topology, offering a transparent and clinically meaningful alternative to conventional black-box models. By modeling patient-specific brain graphs and quantifying their topological properties, the framework provides biologically relevant insights into disease-related alterations in brain connectivity.

Clinically, this model offers a valuable tool for identifying quantifiable biomarkers to support staging and monitoring of Alzheimer’s disease progression. Additionally, its compatibility with privacy-preserving frameworks such as federated learning makes it particularly well-suited for multi-center collaborative studies, where patient data confidentiality is essential. By integrating classification accuracy, interpretability, and secure collaborative infrastructure, this graph-based framework represents a scalable and practical approach for advancing Alzheimer’s disease detection and management. Future work will focus on extending this framework to multimodal imaging and expanding its application to other neurodegenerative conditions.

## Data Availability

The dataset used in this study was obtained from Kaggle and is based on the OASIS-1 dataset. It is available at: https://www.kaggle.com/code/anirudhasutar/alzhimer-disease-detection-oasis-dataset/input. The original OASIS dataset can also be accessed from https://www.oasis-brains.org/. The code that are implemented in this study paper are archived in https://github.com/Gnbhavi/Brain_graph_analysis.git.
